# Supplementary material for: Machine learning and deep learning-based approach to categorize Bengali comments on social networks using fused dataset
Source: PLoS One. 2024 Oct 3;19(10):e0308862. doi: 10.1371/journal.pone.0308862 (PMC11449346; doi:10.1371/journal.pone.0308862)
Supplement: Attachment — Submitted filename: Response to Reviewers.docx [file pone.0308862.s001.docx]

**Manuscript Number:** PONE-D-24-03168

**Title:** Machine Learning and Deep Learning-Based Hybrid Strategy to Categorize Bengali Comments on Social Networks Using a Fused Dataset.

**Journal:** PLOS ONE.
 
Dear Editor,

Thank you for allowing a resubmission of our manuscript, with an opportunity to address the reviewers’ comments. Below is the point-by-point response to the comments, changes are made according to the reviewers' comments and a revised version of the manuscript is attached. All modifications to the paper are highlighted using RED Color.

**Response to reviewer’s comments:**

**Reviewer #1:**

**Concern 1.** The title should be reviewed, especially the words "hybrid strategy" and "a" should be removed before the words "Fused Dataset.

**Author response:**

Thank you for your valuable feedback. We appreciate your thorough review of our manuscript. We will revise the title by removing the words "hybrid strategy" and "a" before "Fused Dataset" to enhance its precision and conciseness.

**Author Action:**

Machine Learning and Deep Learning-Based Approach to Categorize Bengali Comments on Social Networks Using Fused Dataset

**Concern** 2. The authors used so many acronyms first without given providing the full meaning such as CNN, LSTM, TF-IDF, NN, ML, DL, etc. Make sure that these acronyms are defined first, then you can use the acronym later.

**Author response:**

Thank you for your valuable feedback on our manuscript. We have ensured that these acronyms are defined before their subsequent use in the document. We also add a section with the acronyms.

**Author Action:**

| **AdaBoost** | Adaptive Boosting | **LightGBM** | Light Gradient-Boosting Machine |
| --- | --- | --- | --- |
| **ANN** | Artificial Neural Network | **MLP** | Multi-Layer Perceptron |
| **BiGRU** | Bidirectional Gated Recurrent Unit | **MNB** | Multinomial Naive Bayes |
| **BERT** | Bidirectional Encoder Representations from Transformers | **NLP** | Natural Language Processing |
| **Bi-LSTM** | Bidirectional Long Short-Term Memory | **RF** | Random Forest |
| **CLSTM** | Convolutional Long Short-Term Memory | **RNN** | Recurrent Neural Network |
| **CNN** | Convolutional Neural Network | **SN** | Social Networks |
| **DT** | Decision Tree | **SA** | Sentiment Analysis |
| **DNN** | Deep Neural Network | **SVM** | Support Vector Machine |
| **ET** | Extra Tree | **SGD** | Stochastic Gradient Descent |
| **IHT** | Instance Hardness Threshold | **TF-IDF** | Term Frequency-Inverse Document Frequency |
| **KNN** | K-Nearest Neighbor | **XGBoost** | Extreme Gradient Boosting |
| **LR** | Logistic Regression |  |  |

**Concern** 3. Some Literature Reviews (LR) are not based on the Bengali Language, I think, these LRs should be removed.

**Author response:**

Thanks for your suggestion. As per your suggestion, to better align our literature review with a focus on the Bengali language, we can consider removing all non-Bengali language literature reviews. However, in doing so, it's essential to ensure that we maintain the depth and breadth of our research. We can aim to replace these reviews with relevant literature in the Bengali language, thus creating a more comprehensive and inclusive review that aligns with our research objectives.

**Author Action:**

…………...

However, utilizing an encoder-decoder-based LSTM network, Das et al. [11] proposed a different approach for identifying instances of hateful language in Bengali. To address the various classes hateful language issue, they employed TF-IDF, word2Vec, and a 1D CNN model in a network using LSTM. An encoder-decoder machine learning model a well-liked NLP tool was presented in this paper as a way to categorize feedback submitted by users in Bengali on profiles	on Facebook.

Eshan et al. [12] also examined a variety of machine learning algorithms, including Random Forest, multinomial Naïve Bayes, Support Vector Machine with linear, Radial Basis Function (RBF), Polynomial, and Sigmoid kernels. They also contrasted these algorithms with characteristics utilizing single-word, bigram, and trigrams, which are used by Count Vectorizer and Tfidf Vectorizer, but found that the SVM Linear kernel produced the most effective outcomes.

A Gated Recurrent Unit (GRU) model was developed by Ishmam et al. [13] to classify feedback from customers on social media sites. 5126 pieces of Bengali feedback were gathered for the purpose of research, and they were divided into five categories: political discourse, spiritual remarks, encouragement, insults, and discrimination towards race and religion. The GRU structure recognized hateful speech with an accuracy of 70.10%.

………………….

Utilizing machine learning techniques like NB, J48, SVM, as well as KNN, Akhter et al. [20] completed an analogous binary categorization assignment to identify Bengali cyberbullying comments. A collection of 2400 Bengali comments classified as either attacked or not was used in their tests. They used the TF-IDF as an extractor of features to teach the SVM classification algorithm, and they were able to achieve 97% accuracy. Nevertheless, multi-class SA was not present in the experiment because it tended toward categorization into binary categories.

…………..

11. Das, A. K., Al Asif, A., Paul, A., & Hossain, M. N. (2021). Bangla hate speech detection on social media using attention-based recurrent neural network. *Journal of Intelligent Systems*, *30*(1), 578-591.
12. Eshan, S. C., & Hasan, M. S. (2017, December). An application of machine learning to detect abusive bengali text. In *2017 20th International conference of computer and information technology (ICCIT)* (pp. 1-6). IEEE.
13. Ishmam, A. M., & Sharmin, S. (2019, December). Hateful speech detection in public facebook pages for the bengali language. In *2019 18th IEEE international conference on machine learning and applications (ICMLA)* (pp. 555-560). IEEE.

……..

20. Akhter, S. (2018, December). Social media bullying detection using machine learning on Bangla text. In *2018 10th International Conference on Electrical and Computer Engineering (ICECE)* (pp. 385-388). IEEE.

**Concern** 4. A comparison table for the deep learning algorithms used in the study should be provided for the ROC CURVE Matric as well.

**Author response:**

Thank you for your suggestion. We appreciate your emphasis on the importance of providing a comparison table for the deep learning algorithms used in the study, including the ROC curve metric. In response to your concern, we will include a comparison table specifically for the deep learning algorithms, incorporating the ROC curve metric. This addition will provide a more comprehensive comparison of the performance of these algorithms in our study. Thank you for your valuable feedback, which will help enhance the quality of our study.

**Author Action:**

-----------------------------

Table 5 shows the AUC scores for multiple machine learning models over various kinds of detection. Each model executed extremely well for identifying non-bully, troll, sexual, religious, and threat content, via AUC scores near or equal to 1.00. However, AdaBoost along with K-NN performed slightly worse in some classes, with AUC values spanning 0.59 to 0.99. Overall, the results show that the models are highly effective, with the majority accomplishing near-perfect AUC scores across all classes.

Table 5. ML models AUC score list.

| **Classifiers** | **AUC score** | | | | |
| --- | --- | --- | --- | --- | --- |
|  | **Not Bully** | **Troll** | **Sexual** | **Religious** | **Threat** |
| LR | 1.00 | 1.00 | 1.00 | 1.00 | 1.00 |
| XGBoost | 1.00 | 1.00 | 1.00 | 1.00 | 1.00 |
| AdaBoost | 0.68 | 0.68 | 0.68 | 0.99 | 0.59 |
| RF | 1.00 | 1.00 | 1.00 | 1.00 | 0.99 |
| K-NN | 0.97 | 0.96 | 0.93 | 0.94 | 0.93 |
| Bagging | 0.86 | 0.78 | 0.82 | 0.98 | 0.83 |
| MLP | 1.00 | 1.00 | 1.00 | 1.00 | 1.00 |
| SGD | 1.00 | 1.00 | 1.00 | 1.00 | 1.00 |
| Stacking | 1.00 | 1.00 | 1.00 | 1.00 | 1.00 |
| Voting | 1.00 | 1.00 | 1.00 | 1.00 | 1.00 |

-----------------------------------

Table 7 compares the AUC scores of multiple deep learning models that identify different types of content. BiGRU, CNN, CLSTM, DNN, along with RNN all had high AUC ratings, indicating superior performance across classes. While the AUC scores differ slightly between models and classes, they consistently outperform in detecting non-bully, troll, sexual, religious, or threat content.

Table 7. AUC score comparison table for all the deep learning algorithms.

| **Classifiers** | **AUC score** | | | | |
| --- | --- | --- | --- | --- | --- |
|  | **Not Bully** | **Troll** | **Sexual** | **Religious** | **Threat** |
| BiGRU | 0.99 | 0.97 | 0.99 | 1.00 | 0.98 |
| CNN | 0.98 | 0.98 | 0.99 | 1.00 | 0.98 |
| CLSTM | 0.98 | 0.97 | 0.99 | 0.99 | 0.98 |
| DNN | 0.98 | 0.97 | 0.99 | 0.99 | 0.98 |
| RNN | 0.98 | 0.97 | 0.99 | 0.99 | 0.98 |

---------------------

Table 9. Comparison of the proposed model with previous investigations

| **References** | **Feature Extraction Technique** | | | **Dataset Size** | **Classes** | **Accu-racy** | **Precession** | **Recall** | **F1 Score** | **AUC score** |
| --- | --- | --- | --- | --- | --- | --- | --- | --- | --- | --- |
| **Haque et al. [5]** | TF-IDF | Count Vectorizer | Word Embedding | 42,036 | 4 | 85.80 | 86.00 | 85.00 | 86.00 | -- |
| **Das et al. [11]** | TF-IDF | -- | Word Embedding | 7,425 | 7 | 77 | -- | -- | -- | -- |
| **Eshan et al. [12]** | -- | -- | -- | 2,500 | -- | 75 | -- | -- | -- | -- |
| **Ishmam et al. [13]** | -- | -- | Word2Vec  Embedding | 5,126 | 6 | 70.10 | 68.00 | 70.00 | 69.00 | -- |
| **Ahmed et al. [14]** | -- | -- | Word Embedding & Tokenization | 44,001 | 2 | 87.91 | 90.00 | 75.00 | 82.00 | -- |
|  |  |  |  |  | 5 | 85.00 | 85.00 | 85.00 | 84.00 | -- |
| **Ahmed et al. [15]** | TF-IDF | -- | Word Embedding | 12,000 | 2 | 80.00 | 80.00 | 80.00 | 80.00 | 0.50 |
| **Emon et al. [16]** | TF-IDF | -- | Word Embedding | 4,700 | 7 | 82.20 | 83.00 | 82.00 | 82.00 | -- |
| **Mahmud et al. [17]** | TF-IDF |  | -- | 3,000 | 2 | 97.00 | 95.00 | 94.00 | 95.00 | -- |
| **M. Khan et al. [18]** | TF-IDF |  | -- | 63,000 | 7 | 62.00 | -- | -- | 58.00 | -- |
| **Romim et al. [19]** | TF-IDF | -- | Word Embedding | 50,314 | 2 | -- | -- | -- | 86.78 | -- |
| **Akhter et al. [20]** | TF-IDF | -- | -- | 2,400 | -- | 97.73 | 98.00 | -- | 98.00 | 0.54 |
| **Akhter et al. [21]** | TF-IDF | -- | -- | 44,001 | 5 | 98.82 | 98.86 | 98.89 | 98.88 | -- |
|  |  |  |  |  | 2 | 98.57 | 98.58 | 98.56 | 98.56 | 0.997 |
| **Our Work** | TF-IDF | Count Vectorizer | Tokenization | 94,000 | 5 | 99.34 | 99.34 | 99.34 | 99.34 | -- |
|  |  |  |  |  | 2 | 99.41 | 99.41 | 99.41 | 99.41 | 1.00 |

**Concern** 5. In the discussion, three (3) LR were only compared with your findings, what about the remaining LR? It should be compared as well.

**Author response:**

Thank you for your concern. We acknowledge the oversight in the discussion where only three LR were compared with our findings. We appreciate your attention to detail and apologize for the incomplete comparison. In response, we will update the discussion section to include a comprehensive comparison with all LR mentioned in the study. This will provide a more thorough analysis of our findings in relation to the existing literature. We appreciate your valuable feedback and the opportunity to improve the quality of our study.

**Author Action:**

Haque et al. [5] utilized various models including SVC, SGD, RF, LR, MNB, DT, CLSTM, BiGRU, BiLSTM, and LSTM, achieving an accuracy of 85.80% with a dataset size of 42,036. Their limitation was lower detection accuracy. Das et al. [11] employed RNN, attention mechanism, LSTM, GRU, and CNN, achieving an accuracy of 77% with a dataset size of 7,425. They faced challenges with lower detection accuracy and limited dataset coverage. Eshan et al. [12] used RF, MNB, and SVM, achieving an accuracy of 75% with a dataset size of 2,500. They also faced limitations in accuracy and dataset coverage. Ishmam et al. [13] employed GRU, Adaboost, RF, NV, and SVC, achieving an accuracy of 70.10% with a dataset size of 5,126. They encountered limitations in accuracy and dataset coverage. Ahmed et al. [14] applied Random Forest, SVM, KNN, Naïve Bayes, and a hybrid neural network (CNN-SVM), achieving an accuracy of 87.91% for CNN-SVM and 85% for SVM with a dataset size of 44,001. Their limitations included being limited to specific categories and having a dataset with limited accuracy. Ahmed et al. [15] utilized MNB, SVM, LR, XGBoost, CNN, LSTM, BLSTM, and GRU, achieving an accuracy of 80% for MNB with a dataset size of 12,000. They faced limitations in accuracy and dataset coverage. Emon et al. [16] employed LinearSVC, LR, MNB, RF, ANN, RNN, and LSTM, achieving an accuracy of 82.20% with a dataset size of 4,700. They faced limitations due to a relatively small dataset and lower detection accuracy. Mahmud et al. [17] utilized LR, MB, DT, RF, SVM, AdaBoost, GB, SGD, ET, KNN, and MLP, achieving an accuracy of 97% with a dataset size of 3,000. They faced limitations due to a relatively small dataset and a limited preprocessing approach. M. Khan et al. [18] employed SVM, NB, RF, KNN, and NN, achieving an accuracy of 62% with a dataset size of 63,000. Their limitations included lower detection accuracy and only 2% of data labeled for the "Religious" class. Romim et al. [19] utilized Bi-LSTM, achieving an F1-score of 86.78% with a dataset size of 50,314. Their study was limited to hate speech detection in Bengali language, and they did not discuss the potential impact of the dataset. Akhter et al. [20] utilized NB, J48, SVM, and KNN, achieving an accuracy of 97.73% with a dataset size of 2,400. They faced limitations in accuracy and dataset coverage. Akhter et al. [21] employed DT, RF, LR, and MLP, achieving an accuracy of 98.82% for MLP and 98.57% for LR with a dataset size of 44,001. They did not use DL models, and their models' execution time was high.

**Reviewer #2:**

The paper discusses the impact of social media on personal issues and the need to address online harassment. The study uses machine learning and natural language processing to analyze Bengali comments for harassment detection, employing various models such as MLP, K-NN, XGBoost, etc., and DL frameworks like DNN, CNN, C-LSTM, and BiLSTM. The hybrid model (MLP+SGD+LR) showed the best performance with 99.34% accuracy, precision, and F1 score for multi-label classification and 99.41% accuracy for binary classification. However, here are some suggestions to improve the quality of the manuscript.

**Concern 1.** Enhance Figure Descriptions: Add more detailed captions to figures to explain their relevance and findings more clearly.

**Author response:**

Thank you for your constructive feedback regarding the figure descriptions. We acknowledge your suggestion to enhance them by adding more detailed captions to explain their relevance and findings more clearly. We will diligently work on improving the clarity and comprehensibility of our figure descriptions to better convey the significance of our findings.

**Concern** 2. Clarification of Technical Terms: Provide a glossary or a more detailed explanation of technical terms and acronyms for readers not familiar with the field.

**Author response:**

Thank you for your valuable suggestion. As per your suggestion, we have revised the manuscript to include a glossary that provides a more detailed explanation of technical terms and acronyms used throughout the paper. This will help readers who may not be familiar with the field to better understand the content. We appreciate your input, and we believe that this addition will enhance the clarity and accessibility of our work.

**Author Action:**

| **AdaBoost** | Adaptive Boosting | **LightGBM** | Light Gradient-Boosting Machine |
| --- | --- | --- | --- |
| **ANN** | Artificial Neural Network | **MLP** | Multi-Layer Perceptron |
| **BiGRU** | Bidirectional Gated Recurrent Unit | **MNB** | Multinomial Naive Bayes |
| **BERT** | Bidirectional Encoder Representations from Transformers | **NLP** | Natural Language Processing |
| **Bi-LSTM** | Bidirectional Long Short-Term Memory | **RF** | Random Forest |
| **CLSTM** | Convolutional Long Short-Term Memory | **RNN** | Recurrent Neural Network |
| **CNN** | Convolutional Neural Network | **SN** | Social Networks |
| **DT** | Decision Tree | **SA** | Sentiment Analysis |
| **DNN** | Deep Neural Network | **SVM** | Support Vector Machine |
| **ET** | Extra Tree | **SGD** | Stochastic Gradient Descent |
| **IHT** | Instance Hardness Threshold | **TF-IDF** | Term Frequency-Inverse Document Frequency |
| **KNN** | K-Nearest Neighbor | **XGBoost** | Extreme Gradient Boosting |
| **LR** | Logistic Regression |  |  |

**Concern** 3. Consistency in Formatting: Ensure consistency in formatting throughout the paper, including figure labels, table headings, and references, to enhance readability.

**Author response:**

Thanks for your suggestion. As per your suggestion, we have carefully reviewed the formatting of our paper and made necessary adjustments to ensure consistency. This includes ensuring uniform formatting for figure labels, table headings, and references throughout the manuscript. We appreciate your attention to detail, and we believe that these improvements will enhance the overall readability of our paper.

**Concern** 4. Expand Discussion Section: Offer a more comprehensive comparison with existing methods, including advantages and potential limitations of the proposed approach.

**Author response:**

Thank you for your valuable suggestion to expand the discussion section. We appreciate your insight and will certainly work on offering a more comprehensive comparison with existing methods. This will include a detailed analysis of the advantages and potential limitations of the proposed approach. Your input will undoubtedly enhance the quality and depth of our study.

**Author Action:**

Haque et al. [5] utilized various models including SVC, SGD, RF, LR, MNB, DT, CLSTM, BiGRU, BiLSTM, and LSTM, achieving an accuracy of 85.80% with a dataset size of 42,036. Their limitation was lower detection accuracy. Das et al. [11] employed RNN, attention mechanism, LSTM, GRU, and CNN, achieving an accuracy of 77% with a dataset size of 7,425. They faced challenges with lower detection accuracy and limited dataset coverage. Eshan et al. [12] used RF, MNB, and SVM, achieving an accuracy of 75% with a dataset size of 2,500. They also faced limitations in accuracy and dataset coverage. Ishmam et al. [13] employed GRU, Adaboost, RF, NV, and SVC, achieving an accuracy of 70.10% with a dataset size of 5,126. They encountered limitations in accuracy and dataset coverage. Ahmed et al. [14] applied Random Forest, SVM, KNN, Naïve Bayes, and a hybrid neural network (CNN-SVM), achieving an accuracy of 87.91% for CNN-SVM and 85% for SVM with a dataset size of 44,001. Their limitations included being limited to specific categories and having a dataset with limited accuracy. Ahmed et al. [15] utilized MNB, SVM, LR, XGBoost, CNN, LSTM, BLSTM, and GRU, achieving an accuracy of 80% for MNB with a dataset size of 12,000. They faced limitations in accuracy and dataset coverage. Emon et al. [16] employed LinearSVC, LR, MNB, RF, ANN, RNN, and LSTM, achieving an accuracy of 82.20% with a dataset size of 4,700. They faced limitations due to a relatively small dataset and lower detection accuracy. Mahmud et al. [17] utilized LR, MB, DT, RF, SVM, AdaBoost, GB, SGD, ET, KNN, and MLP, achieving an accuracy of 97% with a dataset size of 3,000. They faced limitations due to a relatively small dataset and a limited preprocessing approach. M. Khan et al. [18] employed SVM, NB, RF, KNN, and NN, achieving an accuracy of 62% with a dataset size of 63,000. Their limitations included lower detection accuracy and only 2% of data labeled for the "Religious" class. Romim et al. [19] utilized Bi-LSTM, achieving an F1-score of 86.78% with a dataset size of 50,314. Their study was limited to hate speech detection in Bengali language, and they did not discuss the potential impact of the dataset. Akhter et al. [20] utilized NB, J48, SVM, and KNN, achieving an accuracy of 97.73% with a dataset size of 2,400. They faced limitations in accuracy and dataset coverage. Akhter et al. [21] employed DT, RF, LR, and MLP, achieving an accuracy of 98.82% for MLP and 98.57% for LR with a dataset size of 44,001. They did not use DL models, and their models' execution time was high.

Using a 94,000 dataset, our work used TF-IDF with Count Vectorizer and Tokenization, resulting in an accuracy of 99.34% for the 5-class classification task and 99.41% for the 2-class classification task. In terms of accuracy, our method performs better than current approaches. Compared with other studies, we achieve significantly higher accuracy by using TF-IDF with Count Vectorizer and Tokenization. Furthermore, our approach tackles the drawbacks noted in earlier research, including reduced detection precision, restricted dataset scope, and particular linguistic limitations.

**Concern** 5. It would be beneficial to include statistical tests in the manuscript to provide a more robust analysis of the results. Utilizing tests such as ANOVA, t-tests, or chi-square tests can help to validate the significance of the findings and strengthen the overall credibility of the study.

**Author response:**

Thank you for your suggestion. We appreciate your emphasis on the importance of statistical tests to enhance the robustness of our analysis. We agree that including statistical tests such as ANOVA, t-tests, or chi-square tests can provide a more comprehensive validation of the significance of our findings, thereby strengthening the credibility of our study. In response to your suggestion, we will incorporate ANOVA and chi-square tests into our manuscript to ensure a more robust analysis of the results. Thank you for your valuable feedback, which will undoubtedly improve the quality of our study.

**Author Action:**

The ANOVA test has a p-value of 0.0. Considering the null assumption is true, the p-value is the likelihood of receiving findings from the test that are no less than the same extreme as those recorded throughout the investigation as shown as Table 3. A p-value of 0.0 in this instance suggests that the null theory is strongly refuted by the proof. The ANOVA test's F-value, also known as the F-statistic, is roughly 20.99. The proportion to the overall variability of each group's means how much variance inside the groups is measured by the F-value. Greater variation between the group means in relation to the statistical variability throughout the groups is indicated by a higher F-value. The Chi^2^ test has a p-value of 1.0. In a similar vein, if the null assumption is correct, the p-value here represents the likelihood of receiving test results that are not less than as extreme as those noted during the test. There may not be an important distinction among the noticed and expected frequencies, according to a p-value of 1.0. The Chi^2^ value is roughly 825999.76. The difference between the actual and anticipated frequencies in the scenario table is measured by the Chi^2^ statistic, also known as the Chi^2^-value. The discrepancy between the expected and observed frequencies is quantified. In conclusion, the ANOVA test indicates a substantial variance between the averages of the groups, whereas the Chi-square test shows no statistically significant variance between what was seen and expected frequencies.

Table 3. ANOVA and chi^2^ tests results.

| **Test Name** | **P-Value** | **F-Value** | **Chi^2^-Value** |
| --- | --- | --- | --- |
| ANOVA | 0.0 | 20.99 | -- |
| Chi^2^ | 1.0 | -- | 825999.76 |

**Concern** 6. Extend the literature by adding new and related to consumer electronics citations.

**Author response:**

Thank you for your suggestion. In line with our efforts to better align our literature review with a focus on the Bengali language, we have reviewed the existing citations and identified the need to include more literature related to consumer electronics. While considering your recommendation, we aim to maintain the depth and breadth of our research. Therefore, we will be adding new and relevant citations related to consumer electronics, ensuring that they are in the Bengali language to create a more comprehensive and inclusive review that aligns with our research objectives.

**Author Action:**

…………...

However, utilizing an encoder-decoder-based LSTM network, Das et al. [11] proposed a different approach for identifying instances of hateful language in Bengali. To address the various classes hateful language issue, they employed TF-IDF, word2Vec, and a 1D CNN model in a network using LSTM. An encoder-decoder machine learning model a well-liked NLP tool was presented in this paper as a way to categorize feedback submitted by users in Bengali on profiles	on Facebook.

Eshan et al. [12] also examined a variety of machine learning algorithms, including Random Forest, multinomial Naïve Bayes, Support Vector Machine with linear, Radial Basis Function (RBF), Polynomial, and Sigmoid kernels. They also contrasted these algorithms with characteristics utilizing single-word, bigram, and trigrams, which are used by Count Vectorizer and Tfidf Vectorizer, but found that the SVM Linear kernel produced the most effective outcomes.

A Gated Recurrent Unit (GRU) model was developed by Ishmam et al. [13] to classify feedback from customers on social media sites. 5126 pieces of Bengali feedback were gathered for the purpose of research, and they were divided into five categories: political discourse, spiritual remarks, encouragement, insults, and discrimination towards race and religion. The GRU structure recognized hateful speech with an accuracy of 70.10%.

………………….

Utilizing machine learning techniques like NB, J48, SVM, as well as KNN, Akhter et al. [20] completed an analogous binary categorization assignment to identify Bengali cyberbullying comments. A collection of 2400 Bengali comments classified as either attacked or not was used in their tests. They used the TF-IDF as an extractor of features to teach the SVM classification algorithm, and they were able to achieve 97% accuracy. Nevertheless, multi-class SA was not present in the experiment because it tended toward categorization into binary categories.

…………..

**Concern** 7. Code Availability: Consider adding a section on the availability of code or data used in the study to enhance transparency and reproducibility.

**Author response:**

Thank you for your suggestion. To enhance transparency and reproducibility, we have uploaded all necessary code and data files to a GitHub repository. You can access the repository through the following link. **[** [**GitHub Repository Link**](https://github.com/hasibulhamim2020/PONE-D-24-03168__Machine-Learning-and-Deep-Learning-Based-Hybrid-Strategy/tree/main) **]** This will allow others to review, replicate, and build upon our work more easily.

**Author Action:**

Data Ability: ………………………………………………………………………………………….…… ……………………………………………. The following GitHub repository link contains the combined dataset file and the code for the models: <https://github.com/hasibulhamim2020/PONE-D-24-03168__Machine-Learning-and-Deep-Learning-Based-Hybrid-Strategy> under a CC-BY 4.0 license.

**Again, thank you for your kind cooperation and consideration.**

**Best Regards,**

**Khandaker Mohammad Mohi Uddin**

**Corresponding Author**
