# Supplementary material for: Machine learning and deep learning-based approach to categorize Bengali comments on social networks using fused dataset
Source: PLoS One. 2024 Oct 3;19(10):e0308862. doi: 10.1371/journal.pone.0308862 (PMC11449346; doi:10.1371/journal.pone.0308862)
Supplement: Attachment — Submitted filename: Response to Reviewers.docx [file pone.0308862.s002.docx]

**Manuscript Number:** PONE-D-24-03168R1

**Title:** Machine Learning and Deep Learning-Based Hybrid Strategy to Categorize Bengali Comments on Social Networks Using a Fused Dataset.

**Journal:** PLOS ONE.
 
Dear Editor,

Thank you for allowing a resubmission of our manuscript, with an opportunity to address the reviewers’ comments. Below is the point-by-point response to the comments, changes are made according to the reviewers' comments and a revised version of the manuscript is attached. All modifications to the paper are highlighted using RED Color.

**Response to reviewer’s comments:**

**Reviewer #2:** I have examined the revised manuscript submitted by the author, which addresses the comments and concerns raised in the previous review. The author has made substantial improvements and provided clear clarifications in response to the feedback. I strongly recommend the manuscript to be published in the current form.

**Author response:**

Thank you very much for your thorough examination of our revised manuscript and your positive feedback. We are delighted to hear that you found our revisions satisfactory and that you recommend our work for publication in its current form. Your constructive comments and suggestions have been invaluable in improving the quality of our manuscript. Your encouraging words mean a lot to us and inspire us to continue striving for excellence in our research. Thanks again for your time and effort in reviewing our work.

**Reviewer #3:** The authors has done a great job, however there are few suggession to improve the quality of paper.

**Author response:**

Thank you very much for your positive feedback on our manuscript and for acknowledging our efforts. We are truly grateful for your suggestions to further improve the quality of our paper. Your insights are highly valued, and we will carefully address each point you have raised to enhance our work.We deeply appreciate the time and effort you have invested in reviewing our manuscript and providing us with constructive feedback.

**Concern 1.** In section 2, related work, each sentence is strated with a reference number, it is suggested to use some mix mode.

**Author response:**

Thank you for your valuable feedback. We deeply appreciate the time and effort you have taken to review our manuscript. Regarding your concern about Section 2, "Related Work," we have made revisions to address this issue. Instead of starting each sentence with a reference number, we have adopted a mixed mode of citation. This integration should improve the readability and flow of the section. If there are any further adjustments or additional concerns, please let us know. Thank you once again for your valuable suggestions.

**Author Action:**

**………………………………………………………………………………………………………………………………………..** A binary and multilabel classifier algorithm was presented by Ahmed et al. [14] to recognize abuse statements on Facebook pages. 44,001 user reviews from well-known public Facebook pages were examined for this study and were divided into classes such as non-bully, sexual, threat, troll, and religious. This NN + Ensemble method produced a multilabel classification accuracy of 85% and a binary classification accuracy of 87.91%.

A model for identifying cyberbullying in texts written in Bangla and Romanized was developed by Ahmed et al. [15] using ML and DL techniques. Three social media datasets were produced by their research: one of them for Bangla, another for Romanized Bangla, and one combined dataset. In the combined dataset, the ML algorithm Multinomial Naive Bayes (MNB) achieved an accuracy rate of 80%.

A Bengali-language method for identifying cyberbullying on social media was proposed by Emon et al. [16]. They used 44,001 Bengali comments from Facebook to test different transformer models, such as Bengali BERT, Bengali DistilBERT, and XLM-RoBERTa. Out of all the models, the XLM-RoBERTa model had the highest accuracy rate (85%) and F1 score (86%).

A technique for using ML algorithms to recognize abusive language in Bangla was proposed by Mahmud et al. [17]. Using logistic regression (LR) and annotated translated Bengali corpora, they were able to identify bullying in Bengali with a 97% accuracy rate.

63,000 Bengali Facebook comments from various celebrity pages were compiled by Khan et al. [18] in order to group fans' sentiments toward the celebrity into five categories: happy, excited, upset, shocked, and content. The feature extractor they used to train SVM, NB, RF, KNN, and NN was TF-IDF. They used the SVM classifier to predict a person's attitude toward a celebrity with a 62% accuracy rate. Even though they employed a sizable dataset for their investigation, the dataset's class imbalance issue resulted in a low accuracy score.

The HS-BAN slanderous speech database in Bengali, which has more than 50,000 categorized statements, was made available by Romim et al. [19]. They investigated language features along with artificial neural network-based methods to develop a common detection of hateful speech systems for Bengali. These comparisons demonstrated that sentences incorporating algorithms developed on unofficial papers performed better than those developed on official texts, leading to the Bi-LSTM models outperforming Fast Text casual word implementation with an F1 score of 86.78%.**……………………………………………………………………………………………………………………………….**

**Concern** 2. Add contibution section in the last part of introduction section.

**Author response:**

Thank you for your valuable feedback. We appreciate your thorough review of our manuscript. Regarding your concern about the contributions section, we have added a new contributions section at the end of the introduction. This section outlines the key contributions of our work to provide readers with a clear understanding of the novel aspects and significance of our research. Thank you once again for your valuable suggestions.

**Author Action:**

**………………………………………………………………..**

The main outcomes of the present study paper are as follows:

- A hybrid machine-learning method for detecting online harassment.
- Cleansed data according to several criteria, such as removing punctuation, numerous spaces, particular characters, non-Bengali text, and numbers.
- For the training and testing of the framework, two datasets are combined.
- Count-Vectorizer, along with TF-IDF for ML and tokenization with padding for DL models are used to extract the feature.
- For efficient undersampling, Iterative Hard Thresholding (IHT) was employed.
- K-Fold cross-validation was used to evaluate the model robustly.
- Tested for feature significance and model efficacy using ANOVA and Chi² tests.
- Examined the performance metrics between the ML, DL, and hybrid models to identify the most successful model.
- Created a web application that allows users to interact with the most optimal model.
- Suggested using a mixed machine-learning approach to identify online harassment.

**Reviewer #4:** The paper has been revised thoroughly; however, there are still a few comments:

**Author response:**

Thank you for acknowledging the thorough revisions made to our manuscript. We appreciate your detailed review and the additional comments you have provided. We are committed to addressing these points to further improve the quality of our paper. Your constructive feedback is invaluable, and we sincerely appreciate the time and effort you have dedicated to reviewing our work.

**Concern 1.** Figure 2: Ensure the same font family and size are used consistently across all text elements for uniformity.

**Author response:**

Thank you for your valuable feedback. We deeply appreciate the time and effort you have taken to review our manuscript. Regarding your concern about the font consistency in Figure 2, we have made the necessary adjustments to ensure that the same font family and size are used consistently across all text elements for uniformity, with only the headlines being bold. Please let us know if there are any further adjustments or concerns. Thank you once again for your time and insights.

**Author Action:**

**Concern** 2. Figure 5: Comments before preprocessing should also be translated into English for the reader to understand the preprocessing.

**Author response:**

Thank you for your valuable feedback. We appreciate your thorough review of our manuscript. Regarding your concern about Figure 5 (note: revised menuscript Figure 6), we have carefully translated all comments before preprocessing into English. This adjustment aims to enhance clarity and ensure that readers can fully understand the preprocessing steps involved. We hope these changes meet your expectations. Thank you once again for your valuable insights and guidance.

**Author Action:**

Fig 6. Original and processed text of Bengali Text Dataset.

**Concern** 3. The basic details of the methods, such as Stochastic Gradient Descent, Logistic Regression, Multi-layer Perceptron, Random Forest, Bagging Classifier, Adaptive Boosting, Deep Neural Network, Recurrent Neural Network, Convolutional Neural Network, Long Short-Term Memory, and Bidirectional Gated Recurrent Unit, seem unnecessary and make the paper lengthy. Consider condensing this section to focus on the most relevant methods to streamline the paper. Figures 10 and 11 also seem unnecessary.

**Author response:**

Thank you for your valuable feedback. We deeply appreciate the time and effort you have taken to review our manuscript. Regarding your concern about the detailed descriptions of the methods, we have made this section smaller and edited all method details. We believe that these concise descriptions are essential for ensuring clarity and comprehension of the methodologies used in our work. However, we have removed Figures 10 and 11 as per your suggestion, as they were deemed unnecessary.

If these changes do not meet your expectations, please let us know. We are committed to making any further adjustments required to improve the manuscript. Thank you once again for your insightful and constructive feedback.

**Again, thank you for your kind cooperation and consideration.**

**Best Regards,**

**Khandaker Mohammad Mohi Uddin**

**Corresponding Author**
